# HDAC5 inhibition attenuates ventricular remodeling and cardiac dysfunction

**DOI:** 10.1186/s13023-023-02896-y

**Published:** 2023-09-04

**Authors:** Chenxi Zhu, Zhehao Piao, Li Jin

**Affiliations:** Department of Cardiology, the Second Affiliated Hospital, Yuying Children’s Hospital of Wenzhou Medical University, No. 306 Hualongqiao Road, Wenzhou, Zhejiang 325000 China

**Keywords:** HDAC5, Cardiac hypertrophy, EGR1, MEF2A, Therapies

## Abstract

**Background:**

This study aimed to investigate the role of histone deacetylase 5 (HDAC5) in ventricular remodeling and explore the therapeutic potential of the HDAC5 inhibitor LMK235.

**Methods:**

A transverse aortic constriction (TAC) mouse model and angiotensin II (Ang II)-treated H9C2 cells were used to evaluate the effects of HDAC5 inhibition with LMK235 on ventricular remodeling and cardiac dysfunction. Additionally, the involvement of the extracellular signal-regulated kinase (ERK)/early growth response protein 1 (EGR1) signaling pathway in regulating myocyte enhancer factor 2 A (MEF2A) expression was assessed.

**Results:**

HDAC5 was upregulated in TAC mice and Ang II-treated H9C2 cells, suggesting its involvement in ventricular remodeling and cardiac dysfunction. LMK235 treatment significantly improved cardiac function in TAC mice and attenuated TAC-induced ventricular remodeling and Ang II-induced H9C2 cell hypertrophy. Mechanically, HDAC5 inhibition activated the ERK/EGR1 signaling pathway.

**Conclusions:**

Our findings demonstrate that HDAC5 may suppress the activation of ERK/EGR1 signaling to regulate MEF2A expression and therefore participate in cardiac pathophysiology.

**Supplementary Information:**

The online version contains supplementary material available at 10.1186/s13023-023-02896-y.

## Introduction

Cardiac hypertrophy is characterized by an increase in the size and thickness of cardiac muscle cells [1]. It responds to stressors such as hypertension, heart valve disease, or congenital heart defects. Cardiac hypertrophy could be physiological or pathological hypertrophy. Pathological cardiac hypertrophy is triggered by conditions such as hypertension, myocardial infarction, or genetic mutations, leading to maladaptive remodeling and ultimately heart failure. The development of cardiac hypertrophy involves a complex interplay of molecular, cellular, and hemodynamic factors.

Histone deacetylases (HDACs) were demonstrated to play significant roles in the development and progression of pathological cardiac hypertrophy through their interactions with multiple signaling pathways and transcription factors [2, 3]. For instance, class II HDACs, such as HDAC5, have been found to regulate the activity of myocyte enhancer factor 2 (MEF2) family members, which are crucial transcription factors involved in cardiac growth and remodeling [3, 4]. Inhibition of HDAC activity has been reported to prevent the development of pathological cardiac hypertrophy in animal models, suggesting that targeting HDACs may offer potential therapeutic benefits [5, 6]. Specially, a study demonstrates that sodium butyrate may function to inhibit HDAC5/HDAC6 to attenuate angiotensin (Ang) II-induced cardiac hypertrophy [7], suggesting HDAC5 may be a therapeutic target of cardiac hypertrophy. Also, HDAC5 nuclear export was believed to respond to hypertrophic stimuli, which results in pathologic hypertrophy [8], indicating that HDAC5 inhibition could be against cardiac hypertrophy. A better understanding of the role of HDACs in cardiac hypertrophy could lead to the development of novel therapeutic strategies for the prevention and treatment of heart failure.

Activation of the extracellular signal-regulated kinase (ERK) pathway has been implicated in the pathological cardiomyocyte hypertrophy [9, 10]. ERK is a member of the mitogen-activated protein kinase (MAPK) family, which plays a crucial role in regulating cell growth, proliferation, and differentiation [11]. It was demonstrated that the activation of ERK1/2 led to eccentric cardiac growth, while its inhibition resulted in concentric growth, indicating that ERK signaling is a critical modulator of the hypertrophic response in cardiomyocytes [12]. Early growth response protein 1 (EGR1) is a zinc-finger transcription factor. Its expression relies on Ras-Raf-MEK-ERK1/2 pathway signaling, which is activated by acute vascular injury, and serum-response elements in the EGR-1 promoter [13]. EGR1 regulates the expression of numerous genes and has been associated with the control of cell growth and differentiation, as well as playing a role in cardiac remodeling [14, 15]. EGR1 has been identified as a potent repressor of MEF2A transcriptional activity, which is a key factor in cardiomyocyte hypertrophy [16]. However, the relationship between HDAC5, ERK/EGR1 signaling, and MEF2A in the context of cardiac hypertrophy remains unclear.

In this study, we aimed to investigate the underlying mechanism of HDAC5 in the regulation of the cardiomyocyte hypertrophy. We tested the expression of HDAC5 in ventricular remodeling in a transverse aortic constriction (TAC) mouse model and in H9C2 cells exposed to Ang II. By using LMK235, an HDAC5 inhibitor, we assessed the cardiac function and ventricular remodeling in TAC mice and Ang II-induced hypertrophy in H9C2 cells. The regulatory roles of HDAC5 on ERK/EGR1 signaling pathway and MEF2A expression were explored. Our findings suggest that targeting HDAC5 with LMK235 may have potential therapeutic value in the treatment of cardiac dysfunction and ventricular remodeling.

## Materials and methods

### Experimental animals

All animal studies and operation procedures reported in this study were conducted in accordance with the Guidelines for the Care and Use of Laboratory Animals [17]. The experimental protocols were approved by the Animal Care and Use Committee at Wenzhou Medical University (approval number: wydw2021-0274). Male C57BL/6J mice, aged 6–8 weeks and weighing 25–35 g, were obtained from Zhejiang Vital River Laboratory Animal Technology Co., Ltd (Zhejiang, China).

### The TAC mouse model

The TAC method was performed to establish cardiac hypertrophy as previously reported [18]. Briefly, the mice were anesthetized by continuously inhaling a mixture of 2% isoflurane and oxygen. A 3 mm longitudinal incision was made in the proximal sternum to expose the aortic arch. The aorta was then constricted using a 6 − 0 silk suture ligature tied tightly around a 27-gauge needle, positioned between the innominate artery and the left common carotid artery. Upon removal of the needle, the chest was closed. Sham-operated mice underwent the same surgical procedure, except for the aortic constriction. Following TAC, the mice were maintained at a constant temperature of 37 °C and closely monitored until they were able to move freely.

### Animal grouping and treatment

After a one-week acclimatization period, all C57BL/6J mice were randomly divided into four groups: the sham group underwent sham operation and received vehicle treatment; the sham + LMK235 group underwent sham operation and received LMK235 treatment; the TAC group underwent TAC operation and received vehicle treatment; and the TAC + LMK235 group underwent TAC operation and received LMK235 treatment. The vehicle consisted of 5% DMSO, 5% Tween 80, 40% PEG300, and 50% saline, in accordance with the instructions provided by MCE Co. Ltd. Following the TAC surgery, mice were treated with either LMK235 (MCE China, 5 mg/kg/day) or the vehicle for 14 days.

### Cell culture and treatment

The H9C2 rat ventricular cell line was acquired from BeNa Culture Collection (Suzhou, China) and maintained in Dulbecco’s Modified Eagle Medium (DMEM, Gibco, Grand Island, NY) supplemented with 10% fetal bovine serum and 1% penicillin/streptomycin. Cells were cultured in an incubator set at 37 °C and 5% CO_2_. Ang II was purchased from Sigma-Aldrich (Cat. no. A9525, St. Louis, MO). Cells were exposed to Ang II at a final concentration of 100 nM, followed by the treatment with low (0.1 µM) and high (1.0 µM) concentrations of LMK235 for 24 h.

### Echocardiography

For unbiased reporting, the ultrasound technicians were not informed of the study’s protocol or the details of the animal groups. Echocardiography was performed 14 days after TAC surgery. Parasternal long-axis view, short-axis view at the papillary muscle level, and 2-D guided M-mode images were recorded. Echocardiography in M-mode detected associated parameters, such as ejection fraction (EF)%, fractional shortening (FS)%, and left ventricular internal dimension at diastole/systole (LVIDd/LVIDs). The transmitral left ventricular outflow tract Doppler spectra (E, A) were recorded from an apical four-chamber view.

### Histopathology

Each cardiac specimen was fixed in 10% neutral buffered formalin, followed by embedding in paraffin for light microscopic examination. Sections were prepared at a thickness of 5 μm and stained with either Hematoxylin and Eosin (H&E) or Masson’s trichrome. These stains allowed for the observation of heart morphology and measurement of the fibrotic area.

### Immunofluorescence

Heart tissues were dehydrated in 30% sucrose, embedded in Optimal Cutting Temperature (OCT) compound, and sectioned into 5-µm-thick fixed frozen sections. H9C2 cells were seeded in 6-well culture plates with cell climbing slices and subjected to the indicated treatments. Fixed frozen sections or 4% paraformaldehyde-fixed cells were used, followed by treatment with 0.5% Triton X-100 for permeabilization. The samples were then blocked with 5% bovine serum albumin for 1 h at room temperature and incubated with the corresponding primary antibody at 4℃ overnight. The following day, the corresponding secondary antibodies were added for 2 h in the dark, and 4,6-diamidino-2-phenylindole (DAPI) was used to stain the nucleus. Finally, an anti-fluorescence quenching solution was added, and images were captured under a microscope (Leica, Germany).

### Determination of cardiomyocyte morphology

After washing with phosphate-buffered saline (PBS) three times, cells were fixed in 4% paraformaldehyde for 15 min, followed by permeabilization using 0.5% Triton X-100 for 10 min. The cells were then stained with fluorescein isothiocyanate (FITC)-Phalloidin (CA1680, Solarbio, Beijing, China) for 20 min. After washing with PBS, nuclei were stained with DAPI for 5 min. Images were captured using an orthoscopic microscope, and cardiomyocyte surface area was analyzed using ImageJ software (NIH, Bethesda, MD, USA).

### Western blotting

Total proteins were extracted from the heart and H9C2 cells after treatment using radioimmunoprecipitation assay buffer (Solarbio) containing phenylmethanesulfonyl fluoride (Solarbio) and protease inhibitors (Applygen, Beijing, China) at a ratio of 100:1:1. Protein concentrations were determined using kits from Beyotime (Shanghai, China). The extracted and quantified proteins were separated by sodium dodecyl sulfate–polyacrylamide gel electrophoresis and transferred to 0.22 μm polyvinylidene fluoride membranes. The membranes were then blocked with skimmed milk and incubated with primary antibodies at 4℃ overnight. After incubating with the corresponding secondary antibody for 2 h the next day, protein bands were visualized using a ChemiDoc MP device (Bio-Rad, Hercules, CA). Antibodies against HDAC5 (A0632, ABclonal, Wuhan, China), BNP (A2179, ABclonal), ANP (66160-1-Ig, Proteintech, Rocky Hill, CT), β-MHC (A7564, ABclonal), MEF2A (ab227120, Abcam, Cambridge, MA), EGR1 (22008-1-AP, Proteintech), ERK (4695 S, CST, Danvers, MA), phospho-ERK (4370 S, CST), and GAPDH (10494-1-AP, Proteintech) were used. Finally, ImageJ software (NIH, USA) was employed for the analysis.

### RNA interference

H9C2 cells were transfected with HDAC5 siRNAs (5’-CGACAATGGGAACTTCTTT-3’) for 24 h, and then, to assess their transfection efficiencies, western blotting was performed using an anti-HDAC5 (1:1000) monoclonal antibody. The siRNA targeting rat HDAC5 was designed and synthesized by RiboBio (Guangzhou, China).

### Statistical analysis

The data are presented as the mean ± standard deviation (SD) and were analyzed using GraphPad Prism 7.0 (GraphPad Software, Inc., San Diego, CA). Two-tailed Student’s t-tests were employed for comparisons between two groups, while ANOVA followed by Tukey’s post hoc test was used for comparisons among multiple groups. In this study, a p-value < 0.05 was considered statistically significant.

## Results

### Upregulation of HDAC5 in in ventricular remodeling of TAC mice and H9C2 cells exposed to Ang II

**Fig. 1:**
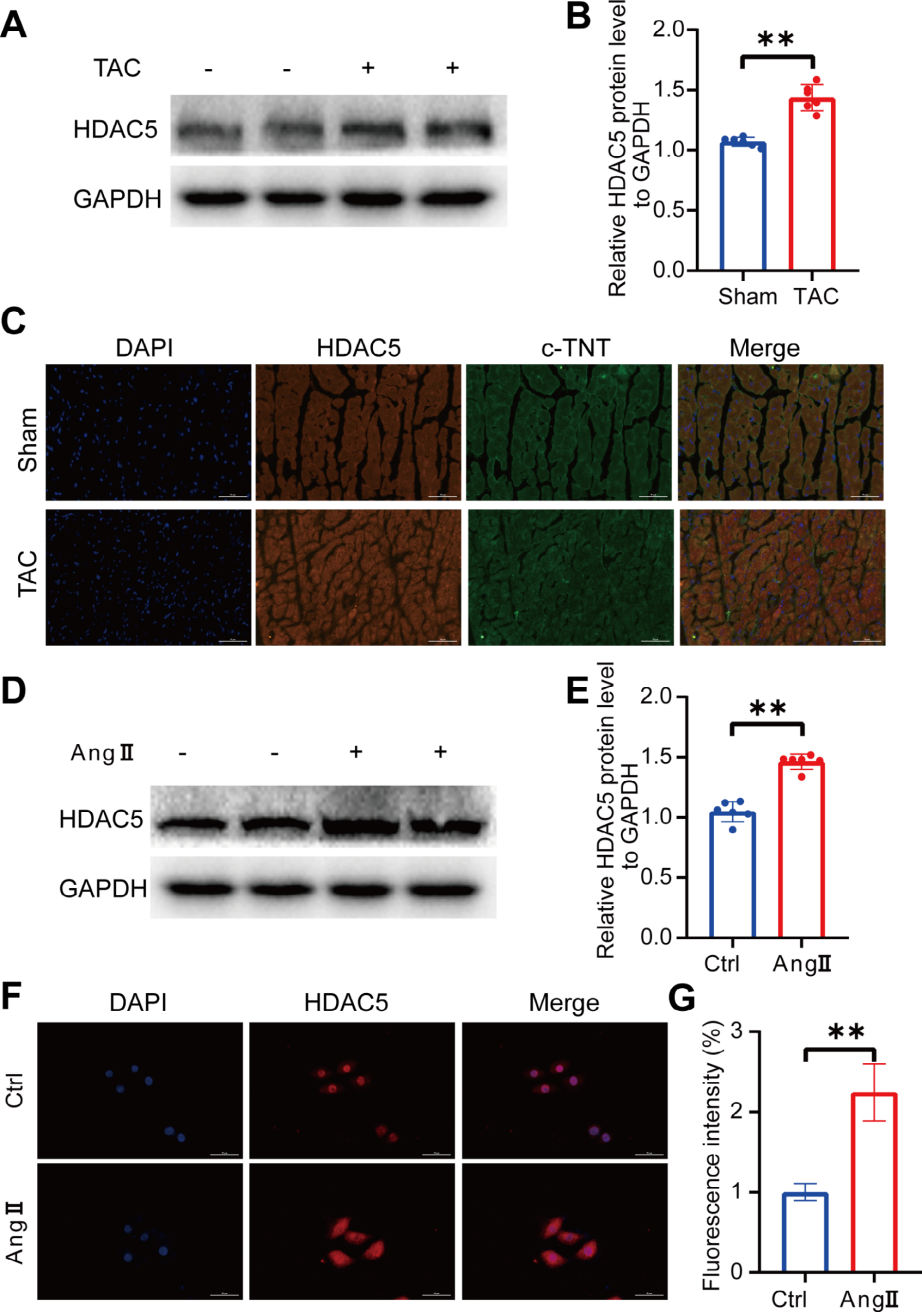
Upregulation of HDAC5 in in ventricular remodeling of TAC mice and H9C2 cells exposed to Ang II (**A-B**) Representative Western blots and quantification showing HDAC5 levels in sham or TAC mice (n = 6 in each group). (**C**) Representative images of immunofluorescence staining of HDAC5 (red), c-TNT (green), and DAPI (blue) in the ventricular remodeling zone of mouse hearts in the sham group and 14 days after TAC. c-TNT: cardiomyocyte marker. Scale bar, 50 μm. (**D-E**) Representative Western blots and quantification showing HDAC5 levels in H9C2 cells in each group (n = 6 in each group). (**F-G**) Representative images of immunofluorescence staining and fluorescence intensity of HDAC5 in H9C2 cells in each group. Scale bar, 50 μm. n > 10 in each group. Data are presented as means ± SD, **P < 0.01

To investigate the role of HDAC5 in ventricular remodeling, we developed a mouse model of TAC. Our results showed a significant increase in HDAC5 protein expression in the hearts of TAC mice compared to control mice, as evidenced by Western blotting analysis (Fig. 1A-B). Moreover, immunofluorescence staining revealed increased colocalization of HDAC5 and cardiac troponin (c-TNT) in the hearts of TAC mice, further confirming the upregulation of HDAC5 during ventricular remodeling (Fig. 1C). Similarly, in Ang II-treated H9C2 cells, we observed a marked increase in HDAC5 protein levels compared to the control group, as determined by Western blotting analysis (Fig. 1D-E). Additionally, immunofluorescence staining data indicated cytoplasmic translocation of HDAC5 in Ang II-treated H9C2 cells (Fig. 1F-G). These results demonstrated that HDAC5 was upregulated during ventricular remodeling, suggesting that HDAC5 may play a role in the development of hypertrophy and heart failure.

### LMK235 ameliorates cardiac dysfunction induced by TAC in vivo

**Fig. 2:**
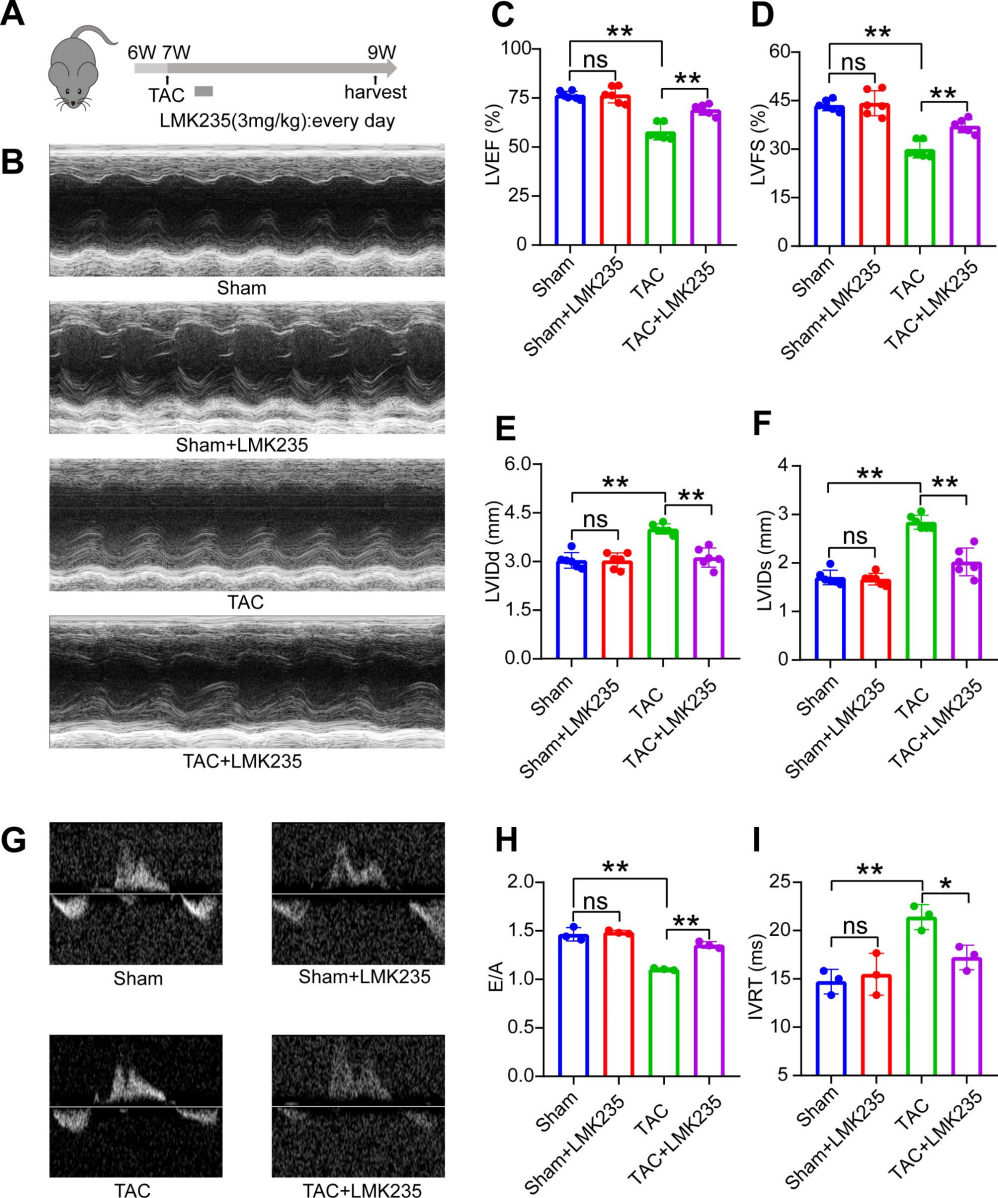
LMK235 ameliorates cardiac dysfunction induced by TAC in vivo (A) Experimental procedure for injecting mice with LMK235. (B) Representative images of M-mode echocardiography of mice in each group. (C, D, E, F) Echocardiographic parameters include left ventricular ejection fraction (LVEF), left ventricular shortening fraction (LVFS), left ventricular end-diastolic diameter (LVIDd), and left ventricular end-systolic diameter (LVIDs) (n = 6 for each group). (G) Representative Doppler echocardiographic images of mitral flow in each group. (H) Mitral inflow velocities (E/A). (I) Isovolumic relaxation time (IVRT) (n = 3 for each group). Data are presented as means ± SD, *P < 0.05; **P < 0.01

To further elucidate the role of HDAC5 in ventricular remodeling and cardiac function, after TAC operation, we used an inhibitor of HDAC5, LMK235, to suppress the expression of HDAC5. No significant weight loss and no obvious abnormal behaviors were observed in the mice treated with LMK235 (data not shown). After TAC operation, we treated mice with LMK235 and sacrificed them 14 days after treatment (Fig. 2A). It was found that TAC mice had a significant decrease in left ventricular ejection fraction (LVEF) and left ventricular fractional shortening (LVFS), as well as a significant increase in left ventricular internal dimension in diastole (LVIDd) and left ventricular internal dimension in systole (LVIDs) (Fig. 2B-F). However, LMK235 treatment for two weeks significantly reversed the increase in LVIDd and LVIDs and improved the decrease in LVEF and LVFS. Additionally, Doppler echocardiography demonstrated that LMK235 reversed the TAC-induced decrease in mitral inflow velocities (E/A) and increase in isovolumic relaxation time (IVRT) (Fig. 2G-I). Comparisons between the sham group and LMK235 treated sham group indicated that LMK235 alone had no significant effect on normal cardiac function. These results suggest that inhibiting HDAC5 expression improves systolic and diastolic dysfunction post-TAC, indicating the potential therapeutic value of LMK235 in ventricular remodeling and cardiac dysfunction.

### LMK235 attenuates TAC-induced ventricular remodeling and Ang II-induced H9C2 cell hypertrophy

**Fig. 3:**
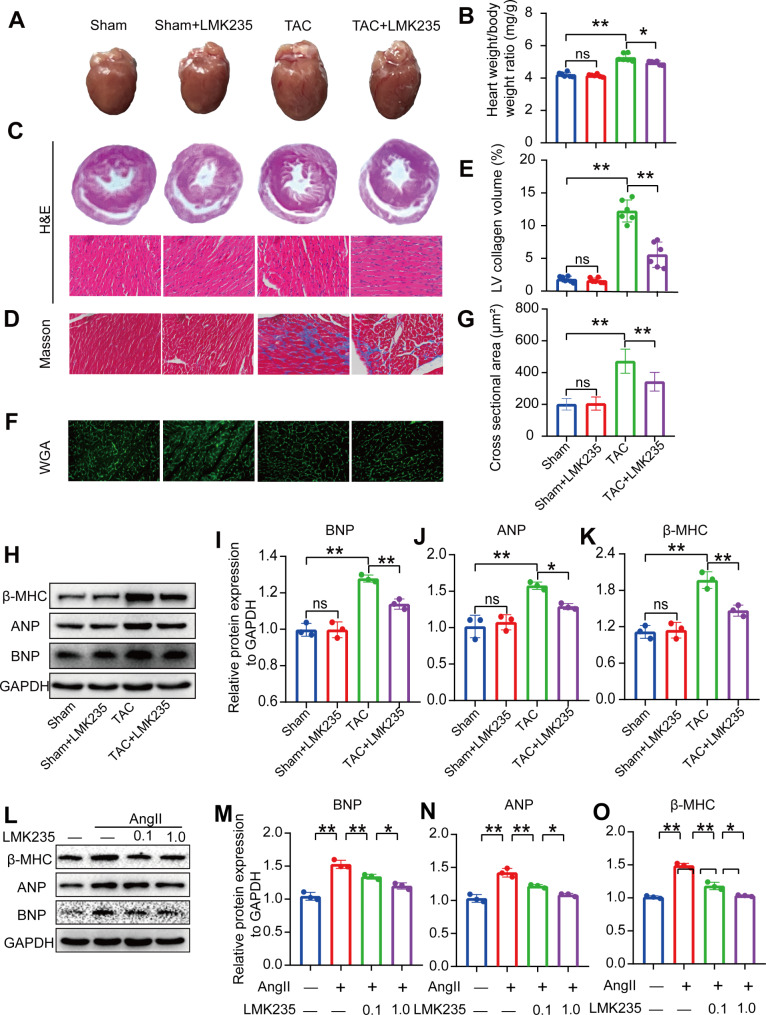
LMK235 attenuates TAC-induced ventricular remodeling and Ang II-induced H9C2 cell hypertrophy (**A**) Representative images of cardiac appearance for each group. (**B**) Heart weight/body weight (HW/BW) (n = 6 for each group). (C) Hematoxylin and eosin staining in horizontal sections of heart tissue. Scale bar, 50 μm. (**D**, **E**) Representative images of Masson’s trichrome staining in each group and statistical analysis of the fibrotic area. Scale bar, 50 μm. (n = 6 for each group). (**F**, **G**) Representative images of WGA staining in each group and statistical analysis of cardiomyocytes cross-sectional area. Scale bar, 50 μm. (n > 50 for each group). (**H**-**K**) Western blot results and statistical analysis of BNP, ANP, and β-MHC expression in mice (n = 3 for each group). (**L**-**O**) Western blot results and statistical analysis of BNP, ANP, and β-MHC expression in H9C2 cells (n = 3 for each group). Data are presented as means ± SD, *P < 0.05; **P < 0.01

The hearts of TAC mice were significantly enlarged compared to the sham group, as demonstrated by stereoscopy, heart weight to body weight ratio (HW/BW), and hematoxylin and eosin (H&E) staining. However, treatment with LMK235 significantly slowed down this change (Fig. 3A-C). Additionally, Masson’s staining revealed interstitial fibrosis and collagen accumulation in the myocardial tissue of TAC mice, which was significantly attenuated by LMK235 treatment (Fig. 3D, E). Furthermore, WGA staining demonstrated a significant increase in the cross-sectional area of cardiomyocytes in TAC mice, which was also significantly attenuated by LMK235 treatment (Fig. 3F, G).

Western blotting analysis revealed significantly higher expression levels of atrial natriuretic peptide (ANP), brain natriuretic peptide (BNP), and β-myosin heavy chain (β-MHC) in the hearts of TAC mice compared to the sham group. However, treatment with LMK235 significantly attenuated the expression of these markers (Fig. 3H-K).

To further confirm these findings, we evaluated the effect of LMK235 on Ang II-induced hypertrophy in H9C2 cells using Western blot analysis. Our results showed that the markers of cardiac hypertrophy were upregulated in the Ang II-treated group, but their expression was significantly downregulated in a concentration-dependent manner following LMK235 treatment (Fig. 3L-O). Moreover, LMK235 treatment inhibited cell hypertrophy induced by Ang II (Supplementary Fig. S1A, B). Interestingly, Ang II stimulation significantly enhanced HDAC9 and MEF2A protein expression in the H9C2 cells. However, LMK235 treatment did not significantly reduce this increase of HDAC9 protein expression, while it successfully reversed the increase of MEF2A protein expression (Supplementary Fig. S2A-2 C). Further, it was found that the level of phosphorylated HDAC5 was dramatically enhanced in in H9C2 cells upon Ang II stimulation (Supplementary Fig. S3A and 3B). We further tested whether LMK235 treatment could affect other HDACs expression. Ang II induced significant up-regulation of all HDACs protein expression. However, LMK235 treatment failed to attenuate these increases of protein expression (Supplementary Fig. S4A-S4H). These results indicate that LMK235 attenuates TAC-induced ventricular remodeling and Ang II-induced H9C2 cell hypertrophy, highlighting the potential therapeutic value of LMK235 in the treatment of cardiac dysfunction.

### LMK235 reverses TAC and Ang II induced HDAC5 expression increase and cytoplasmic translocation

**Fig. 4:**
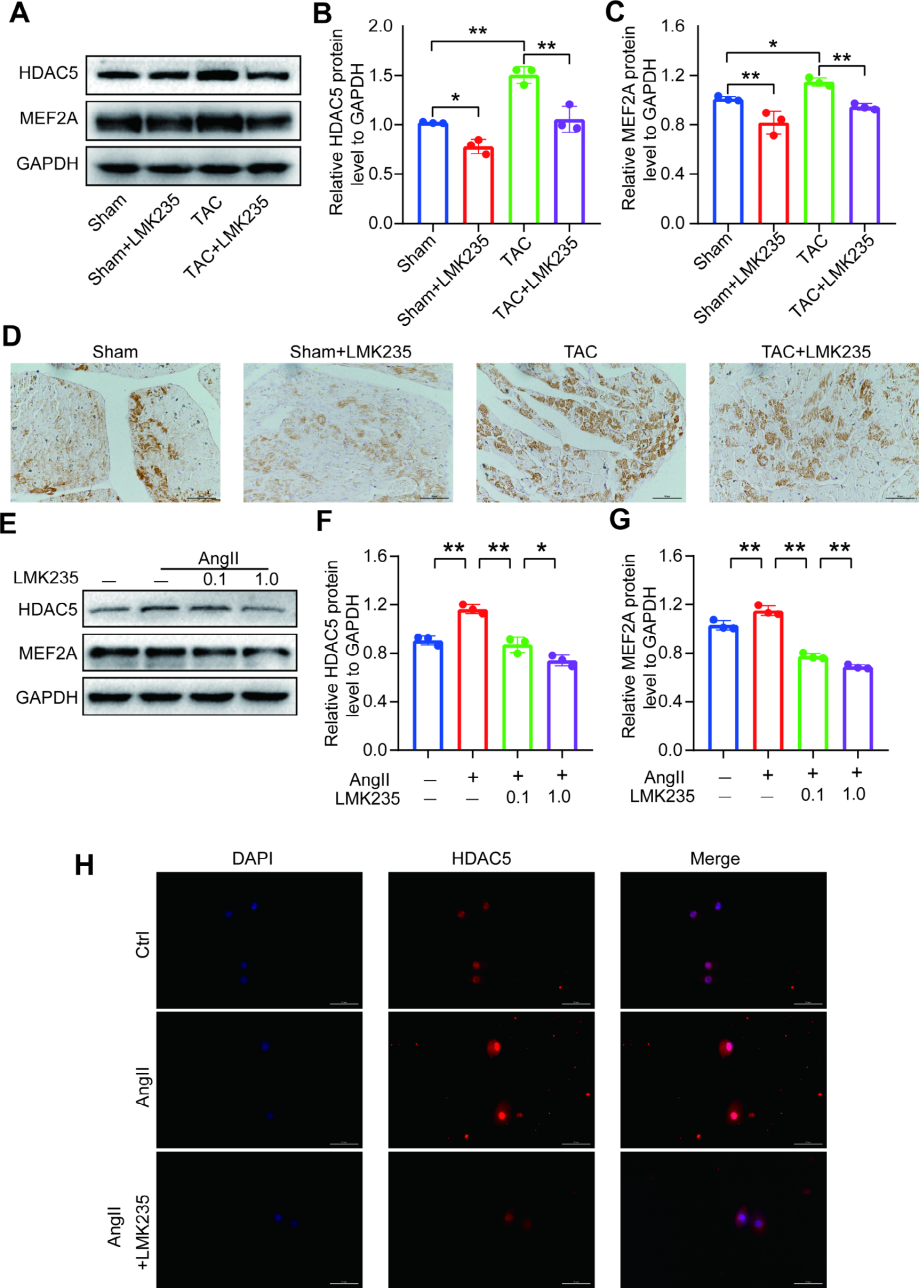
LMK235 reverses TAC and Ang II induced HDAC5 expression increase and cytoplasmic translocation (**A-C**) Western blot results and statistical analysis of HDAC5 and MEF2A expression in mice (n = 3 for each group). (**D**) Representative images of immunohistochemical staining of HDAC5 in each group. Scale bar, 50 μm. (**E-G**) Western blot results and statistical analysis of HDAC5 and MEF2A expression in H9C2 cells (n = 3 for each group). (**H**) Representative images of immunofluorescence staining of HDAC5 in H9C2 cells in each group. Scale bar, 50 μm. Data are presented as means ± SD, *P < 0.05; **P < 0.01

LMK235 significantly reduced the protein level of HDAC5 after TAC surgery (Fig. 4A, B), which was confirmed by immunohistochemistry. However, treatment with LMK235 alone did not have a significant effect on ventricular remodeling (Fig. 3A-G). Our cell experiments demonstrated that HDAC5 expression was upregulated in the Ang II-treated group but was concentration-dependently downregulated by LMK235 treatment (Fig. 4E, F). Immunofluorescence staining of HDAC5 showed a similar expression pattern and cytoplasmic translocation (Fig. 4H).

Furthermore, we found that MEF2A, which was found to be activated by CaMKII/PKD-induced HDAC5 cytoplasmic translocation [19, 20], was significantly upregulated after TAC surgery and Ang II treatment. However, MEF2A expression was downregulated following the inhibition of HDAC5 expression both in vivo (Fig. 4A, C) and in vitro (Fig. 4E, G). These results suggest that HDAC5 may play a role in regulating MEF2A expression and potentially contribute to the early stage of ventricular remodeling.

### HDAC5 inhibition activates ERK/EGR1 signaling to regulate MEF2A expression during cardiomyocyte hypertrophy

**Fig. 5:**
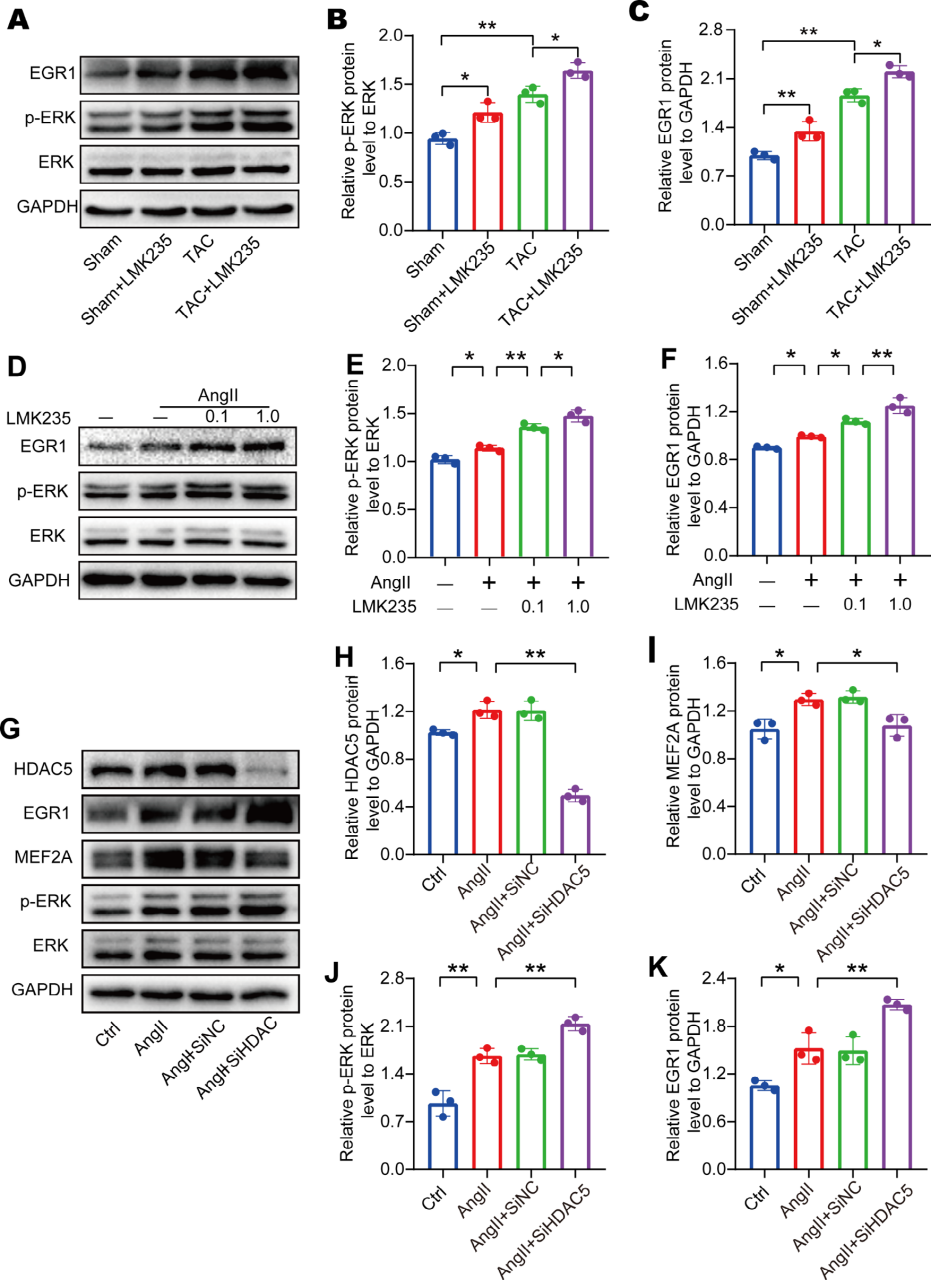
HDAC5 inhibition activates ERK/EGR1 signaling to regulate MEF2A expression during cardiomyocyte hypertrophy (**A-C**) Western blot results and statistical analysis of EGR1, ERK, and p-ERK expression in mice (n = 3 for each group). (**D-F**) Western blot results and statistical analysis of EGR1, ERK, and p-ERK expression in H9C2 cells (n = 3 for each group). (**G-H**) Western blot results and statistical analysis of HDAC5, MEF2A, EGR1, ERK, and p-ERK expression in H9C2 cells after transfection with HDAC5 siRNA (n = 3 for each group). Data are presented as means ± SD, *P < 0.05; **P < 0.01

Recent studies showed that HDAC inhibitors activated the ERK/EGR1 pathway [21]. EGR1, a potent repressor of MEF2A transcriptional activity [16], may play a role in the regulation and control of cardiomyocyte hypertrophy. We found that the ERK/EGR1 signaling was active in TAC mice and was further activated after treatment with the histone deacetylase inhibitor LMK235 (Fig. 5A-C). Immunofluorescence staining also revealed an increase in the colocalization of p-ERK and cardiac troponin (c-TNT) in TAC mice, which was further increased after LMK235 treatment (Supplementary Fig. S5A). In our cell experiments, LMK235 treatment also further activated the ERK/EGR1 signaling (Fig. 5D-F).

To confirm our observations, we performed HDAC5 siRNA transfection in H9C2 cells. As observed in mice, interfering with HDAC5 expression significantly reduced the expression of MEF2A. Moreover, the ERK/EGR1 pathway was significantly activated (Fig. 5G-K). Similar activation of the signaling was observed when cells were transfected with HDAC5 siRNA (Supplementary Fig. S5B-F). These findings suggest that HDAC5 may regulate MEF2A expression by influencing the ERK/EGR1 signaling pathway, which is involved in the progression of myocardial hypertrophy.

## Discussion

In this study, we investigated the role of HDAC5 in ventricular remodeling and its potential as a therapeutic target for the treatment of cardiac dysfunction. Our results demonstrated that HDAC5 was upregulated in both TAC mice and Ang II-treated H9C2 cells, suggesting its involvement in the development of cardiac hypertrophy and heart failure. We found that treatment with LMK235, an HDAC5 inhibitor, ameliorated cardiac dysfunction and attenuated ventricular remodeling in TAC mice and Ang II-induced hypertrophy in H9C2 cells. We also observed that HDAC5 inhibition led to the activation of the ERK/EGR1 signaling pathway, which in turn regulated MEF2A expression.

Consistently with our findings, a recent study showed that the expression of HDAC5 in the hearts of TAC mice increases progressively following TAC [22]. Another study found that Ang II treatment significantly increased HDAC5 mRNA levels in rat cardiac tissues and showed an increase in the active content of HDAC5 [23]. Overexpression of HDAC5 in cardiomyocytes inhibits MEF2 expression and leads to pathological hypertrophy in response to stimuli [3, 24]. On the other hand, silencing of HDAC5 could cause an exaggerated hypertrophic response to pressure overload and spontaneous hypertrophy at an advanced age [4]. In addition, repression of HDAC5 phosphorylation and preventing HDAC5 nuclear export by YY1 overexpression significantly inhibited the increase in cell size and the re-expression of fetal genes associated with pathological cardiac hypertrophy [25].

HDAC inhibitors have been found to effectively attenuate pathological ventricular remodeling and exert protective effects against cardiac hypertrophy by various mechanism such as modulating the acetylation and deacetylation of target genes [26–29]. In this study, we used LMK235, an HDAC5 inhibitor, led to significant improvements in cardiac function in TAC mice, as evidenced by the reversal of systolic and diastolic dysfunction. Moreover, our findings revealed that LMK235 attenuated TAC-induced ventricular remodeling and Ang II-induced H9C2 cell hypertrophy. These results support the potential therapeutic value of LMK235 in ventricular remodeling and cardiac dysfunction.

Our data also showed that HDAC5 inhibition activated the ERK/EGR1 signaling pathway, which in turn regulated MEF2A expression. HDAC was showed to function as repressors of MEF2 [30], and MEF2A was found to be activated after HDAC5 cytoplasmic translocation [19, 20]. MEF2A is a member of the MEF2 family of transcription factors, which also includes MEF2B, MEF2C, and MEF2D. MEF2 has been shown to be a critical regulator of cardiac hypertrophy and remodeling [31, 32]. We found that MEF2A expression was downregulated following the inhibition of HDAC5 expression both in vivo and in vitro. This suggests that HDAC5 may play a role in regulating MEF2A expression and potentially contribute to the early stages of ventricular remodeling.

**Fig. 6:**
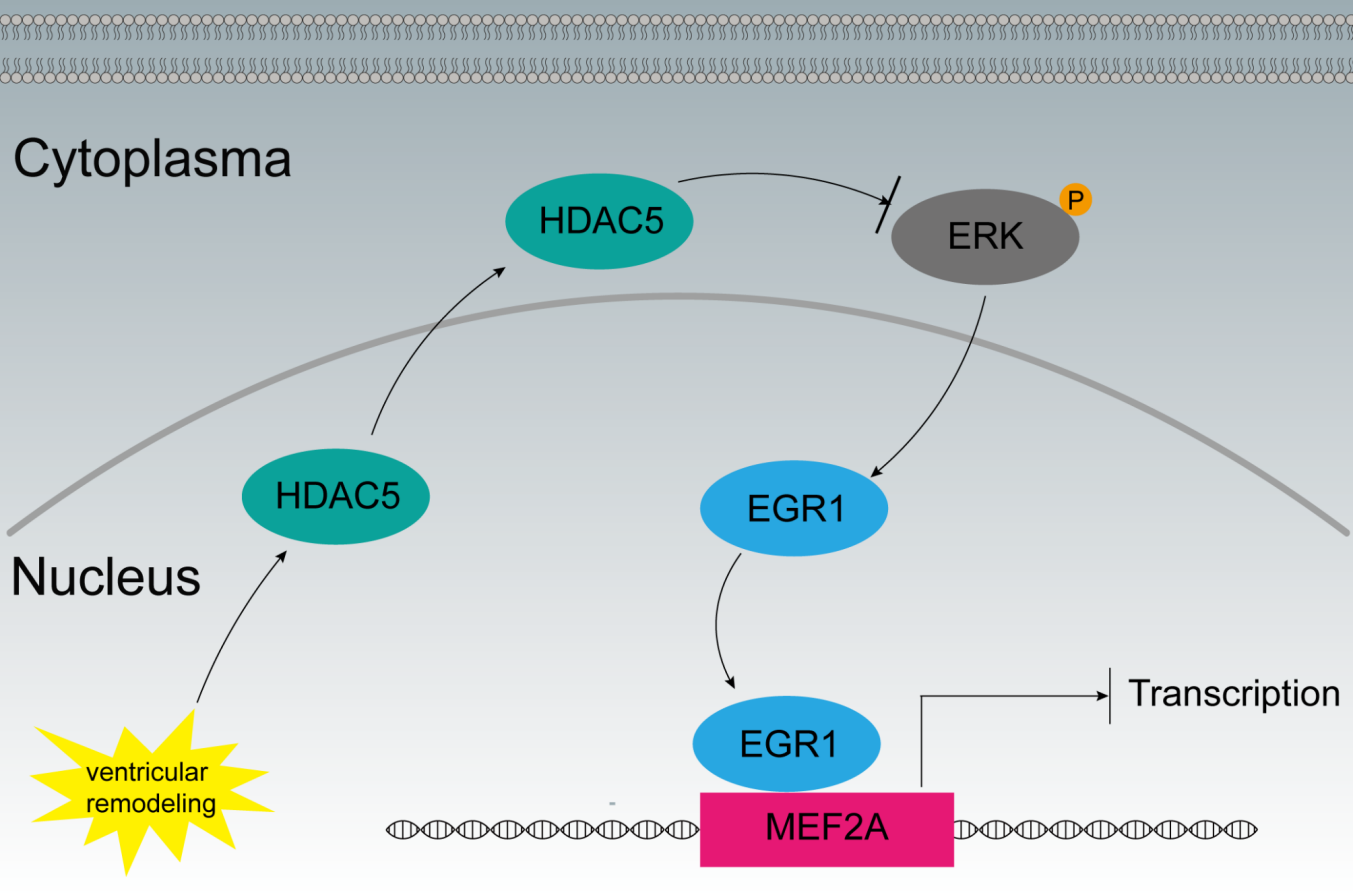
HDAC5 regulates MEF2A expression by influencing the ERK/EGR1 signaling pathway Ventricular remodeling induces cytoplasm translocation of HDAC5. Inhibition of ERK/EGR1 by HDAC5 results in a marked upregulation of MEF2A, which consequently contributes to the promotion of TAC-induced ventricular remodeling and Ang II-induced hypertrophy in H9C2 cells

Previous studies demonstrated a regulatory role of HDAC in ERK signaling [33]. A recent research revealed that HDAC inhibitors stimulated the ERK/EGR1 pathway [21], demonstrating that HDAC represses ERK/EGR1 signaling activation. EGR1 is a potent repressor of MEF2A transcriptional activity [16], and was suggested to play a role in the regulation and control of cardiomyocyte hypertrophy [13]. Our findings indicate that the ERK/EGR1 signaling was active in TAC mice, and its activation was further enhanced following treatment with LMK235. HDAC5 depletion using siRNA in H9C2 cells showed the same results. These findings imply that HDAC5 might regulate MEF2A expression by negatively influencing the ERK/EGR1 signaling pathway. The suppression of ERK/EGR1 by HDAC5 led to a significant overexpression in MEF2A, which in turn promotes TAC-induced ventricular remodeling and Ang II-induced H9C2 cell hypertrophy (Fig. 6). However, future studies are needed to elucidate the precise molecular mechanisms by which HDAC5 modulates MEF2A expression and the ERK/EGR1 signaling pathway. Moreover, further exploration of the potential of HDAC5 inhibition as a therapeutic strategy for the treatment of heart failure and ventricular remodeling is warranted. It is important to note that this study was limited to a single model of cardiac hypertrophy and heart failure. Future research should explore the role of HDAC5 in different models of heart disease to better understand its function and relevance as a therapeutic target.

## Conclusion

In summary, our study provides compelling evidence for the upregulation of HDAC5 in ventricular remodeling of TAC mice and H9C2 cells exposed to Ang II. The use of the HDAC5 inhibitor LMK235 demonstrated its potential therapeutic value in mitigating cardiac dysfunction and attenuating ventricular remodeling. Furthermore, our findings suggest that HDAC5 may regulate MEF2A expression through the ERK/EGR1 signaling pathway, contributing to the progression of myocardial hypertrophy. We provide a better understanding of the molecular mechanisms underlying this interaction may contribute to the development of novel therapeutic strategies targeting HDAC5 and MEF2A for the treatment of heart failure and other cardiovascular diseases.

### Electronic supplementary material

Below is the link to the electronic supplementary material.

**Figure S1**. (A) Representative images of immunofluorescence staining of F-actin in H9C2 cells in each group. (B) The quantitative analysis of cell surface area in each group. Data are presented as means ± SD, **P<0.01. **Figure S2**. The expression of HDAC9 and MEF2A in the H9C2 cells with or without Ang II stimulation, in the presence or absence of LMK235. (A) Western blot analysis was used to determine the protein expression. Relative HDAC9 (B) and MEF2A (C) expression was normalized to GAPDH. Data are presented as means ± SD, *P<0.05; **P<0.01. **Figure S3**. The HDAC5 phosphorylation in H9C2 cells upon Ang-II stimulation. Phosphorylated HDAC5 was determined by Western blot analysis (A), and the relative expression was normalized to GAPDH (B). Data are presented as means ± SD, **P<0.01. **Figure S4**. The expression of HDACs in H9C2 cells upon LMK235 treatment. The protein expression was determined by Western blot analysis (A). Relative expression of HDAC1 (B), HDAC2 (C), HDAC3 (D), HDAC4 (E), HDAC7 (F), HDAC8 (G) and HDAC9 (H) was normalized to GAPDH. Data are presented as means ± SD, *P<0.05, **P<0.01. ns indicates no significant difference. **Figure S5**. (A) Representative images of immunofluorescence staining of p-ERK in each group. Scale bar, 50 μm. (B-F) Western blot results and statistical analysis of HDAC5, MEF2A, EGR1, ERK, and p-ERK expression in H9C2 cells after transfection with HDAC5 siRNA alone (n=6 for each group). Data are presented as means ± SD; **P<0.01.

Supplementary Material 2

## Data Availability

The original data and materials could be obtained upon reasonable request to the corresponding author.

## Abbreviations

HDAC5: Histone deacetylase 5
TAC: Transverse aortic constriction
Ang: II angiotensin II
ERK: Extracellular signal-regulated kinase
EGR1: Early growth response protein 1
