# Supplementary material for: HDAC5 inhibition attenuates ventricular remodeling and cardiac dysfunction
Source: Orphanet J Rare Dis. 2023 Sep 4;18:266. doi: 10.1186/s13023-023-02896-y (PMC10476361; doi:10.1186/s13023-023-02896-y)
Supplement: Supplementary file 1 — Figure S1. (A) Representative images of immunofluorescence staining of F-actin in H9C2 cells in each group. (B) The quantitative analysis of cell surface area in each group. Data are presented as means ± SD, **P<0.01. Figure S2. The expression of HDAC9 and MEF2A in the H9C2 cells with or without Ang II stimulation, in the presence or absence of LMK235. (A) Western blot analysis was used to determine the protein expression. Relative HDAC9 (B) and MEF2A (C) expression was normalized to GAPDH. Data are presented as means ± SD, *P<0.05; **P<0.01. Figure S3. The HDAC5 phosphorylation in H9C2 cells upon Ang-II stimulation. Phosphorylated HDAC5 was determined by Western blot analysis (A), and the relative expression was normalized to GAPDH (B). Data are presented as means ± SD, **P<0.01. Figure S4. The expression of HDACs in H9C2 cells upon LMK235 treatment. The protein expression was determined by Western blot analysis (A). Relative expression of HDAC1 (B), HDAC2 (C), HDAC3 (D), HDAC4 (E), HDAC7 (F), HDAC8 (G) and HDAC9 (H) was normalized to GAPDH. Data are presented as means ± SD, *P<0.05, **P<0.01. ns indicates no significant difference. Figure S5. (A) Representative images of immunofluorescence staining of p-ERK in each group. Scale bar, 50 μm. (B-F) Western blot results and statistical analysis of HDAC5, MEF2A, EGR1, ERK, and p-ERK expression in H9C2 cells after transfection with HDAC5 siRNA alone (n=6 for each group). Data are presented as means ± SD; **P<0.01. [file 13023_2023_2896_MOESM1_ESM.docx]

**Supplementary materials**

**
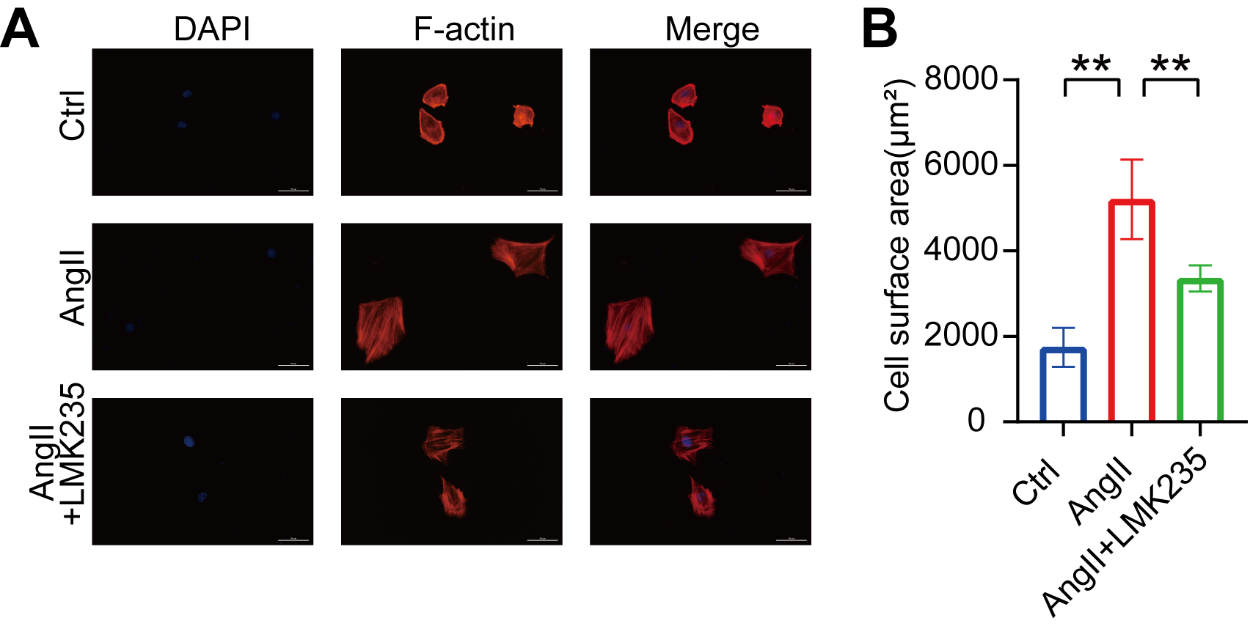
**

**Figure S1.** ﻿(A) Representative images of immunofluorescence staining of F-actin in H9C2 cells in each group. (B) The quantitative analysis of cell surface area in each group. Data are presented as means ± SD, **P < 0.01.


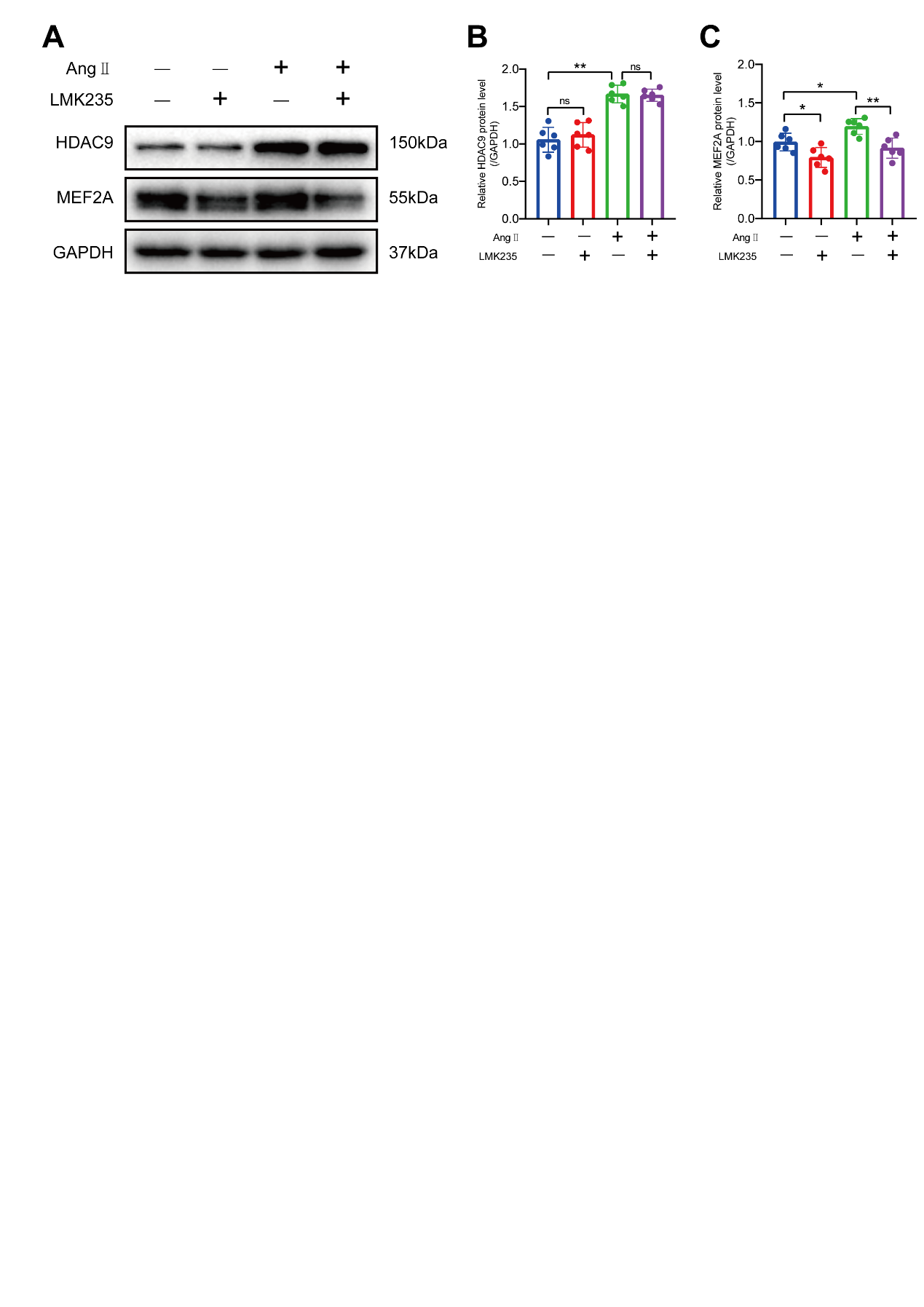


Figure S2. The expression of HDAC9 and MEF2A in the H9C2 cells with or without Ang II stimulation, in the presence or absence of LMK235. (A) Western blot analysis was used to determine the protein expression. Relative HDAC9 (B) and MEF2A (C) expression was normalized to GAPDH. Data are presented as means ± SD, *P < 0.05; **P < 0.01.


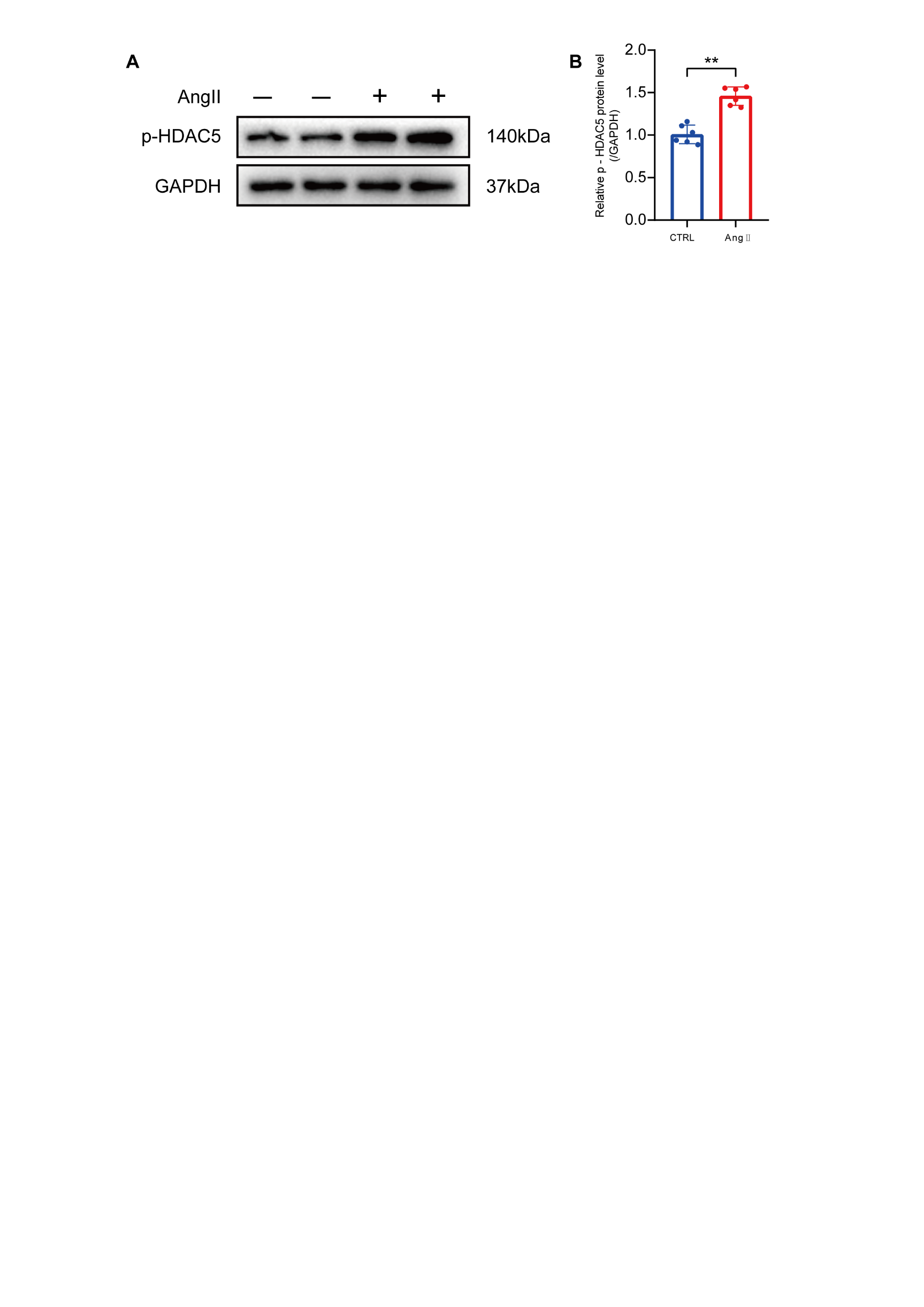


Figure S3. The HDAC5 phosphorylation in H9C2 cells upon Ang-II stimulation. Phosphorylated HDAC5 was determined by Western blot analysis (A), and the relative expression was normalized to GAPDH (B). Data are presented as means ± SD, **P < 0.01.


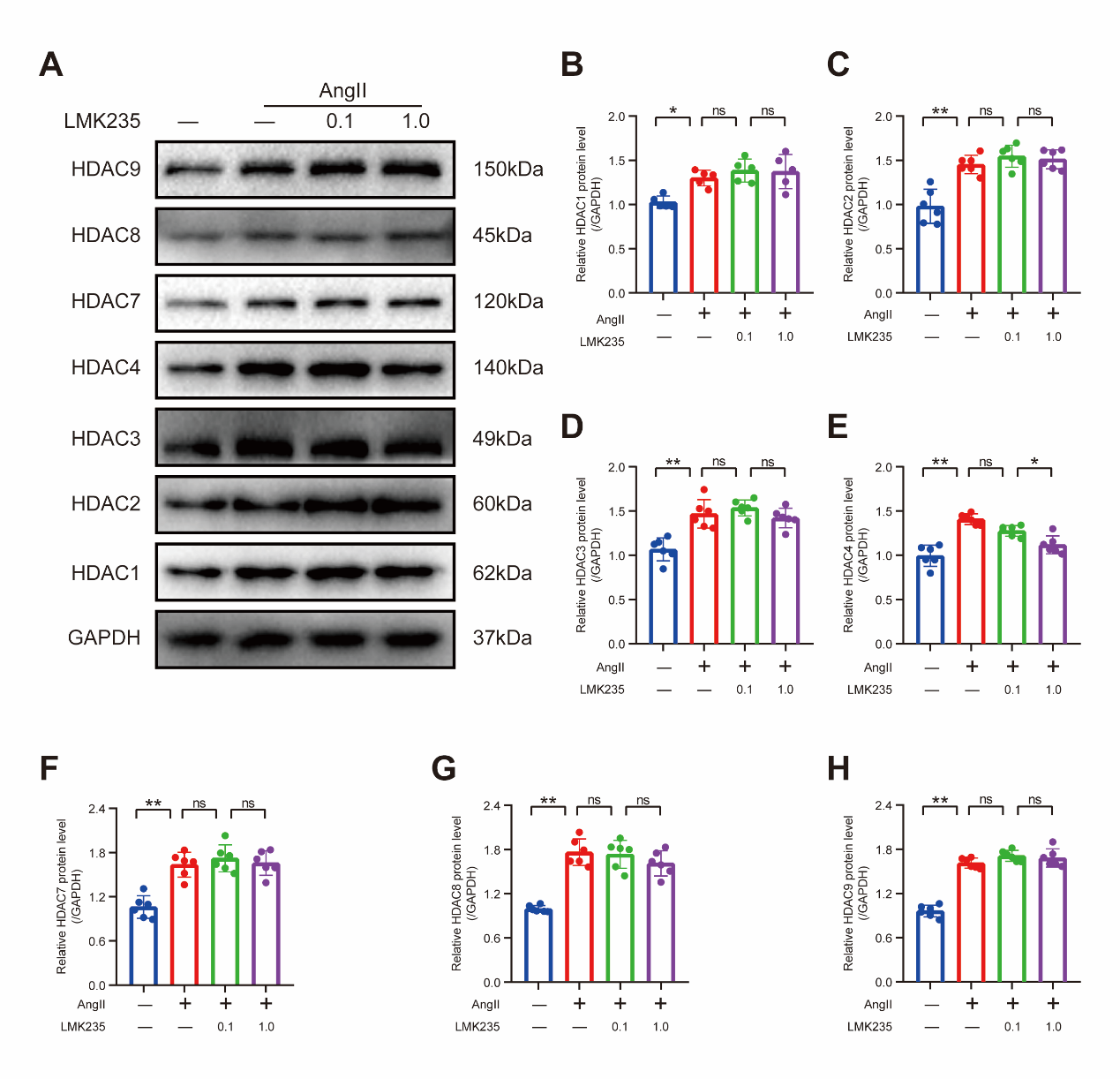


Figure S4. The expression of HDACs in H9C2 cells upon LMK235 treatment. The protein expression was determined by Western blot analysis (A). Relative expression of HDAC1 (B), HDAC2 (C), HDAC3 (D), HDAC4 (E), HDAC7 (F), HDAC8 (G) and HDAC9 (H) was normalized to GAPDH. Data are presented as means ± SD, *P < 0.05, **P < 0.01. ns indicates no significant difference.


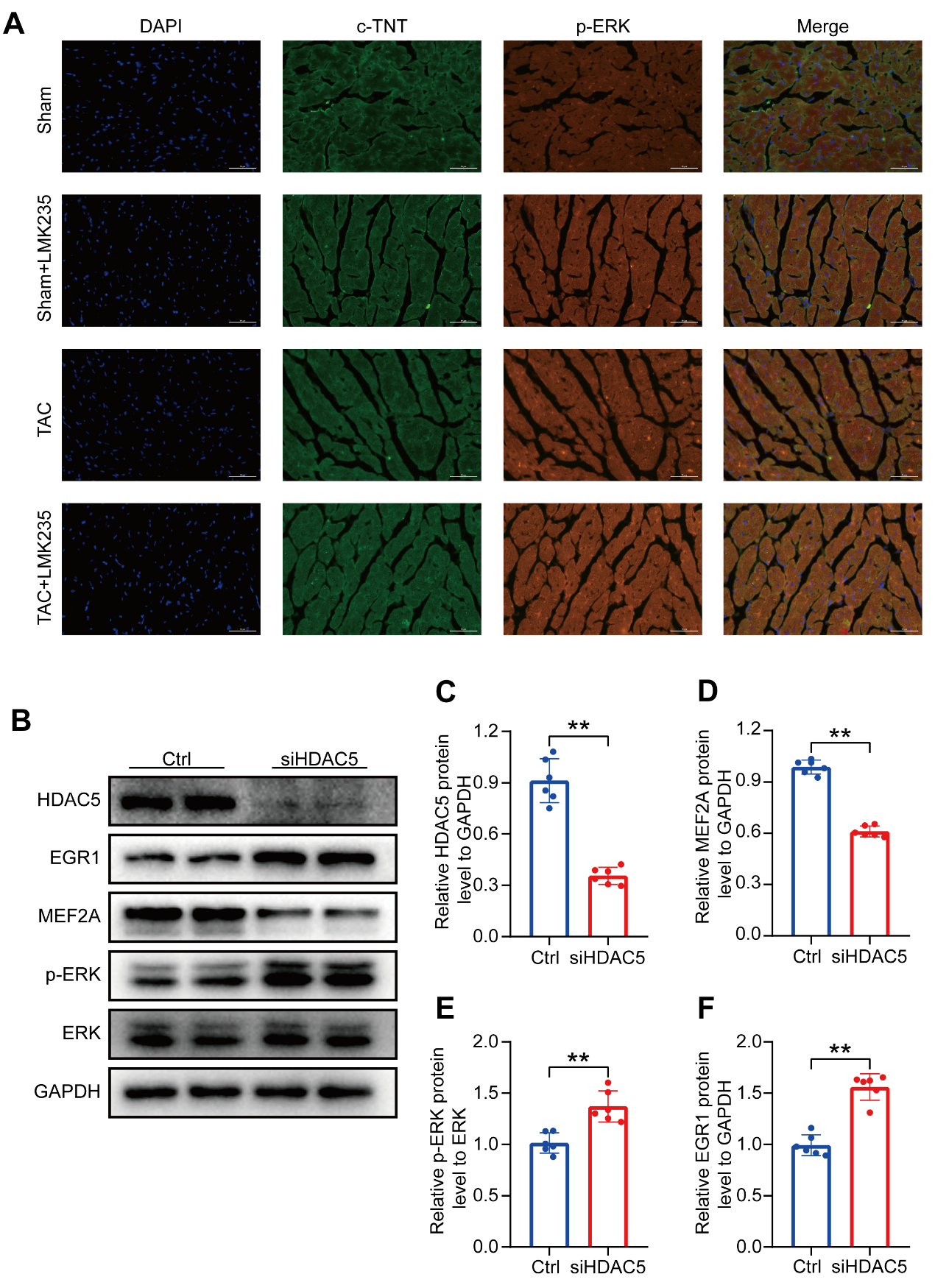


**Figure S5.** (A) Representative images of immunofluorescence staining of p-ERK in each group. Scale bar, 50 µm. (B-F) Western blot results and statistical analysis of HDAC5, MEF2A, EGR1, ERK, and p-ERK expression in H9C2 cells after transfection with HDAC5 siRNA alone (n=6 for each group). Data are presented as means ± SD; **P < 0.01.
