# Supplementary material for: HDAC5 inhibition attenuates ventricular remodeling and cardiac dysfunction
Source: Orphanet J Rare Dis. 2023 Sep 4;18:266. doi: 10.1186/s13023-023-02896-y (PMC10476361; doi:10.1186/s13023-023-02896-y)
Supplement: Supplementary file 2 — Supplementary Material 2 [file 13023_2023_2896_MOESM2_ESM.docx]

**Reviewer #2:**

I appreciate the efforts the authors made to improve the manuscript after Reviewer 1 and Reviewer 2 comments. However, in the process of reviewing the manuscript a mistake has been made and need to be addressed.

HDAC5 in vitro model
After my comments, authors changed the Supplementary materials to provide some of the demanded answers. Unfortunately, along this process data on HDAC5 Knocked Down H9C2 cells has been erased (Version 1, Suppl. Mat. 2 B and D), although no comments on this change are made and siRNA methods are still present in the mats & meths section. 

Therefore, the following question has not clearly been addressed or answered: "it would be interesting to know how HDAC5 Knock Down H9C2 cells behave under Ang-II stimulus"
1. Could the authors provide some more evidence on how the silencing of HDAC5 expression affects MEF2 and HDAC9 expression, with and without Ang-II stimulation?

Response:

Thanks for the comments. Actually, we did not delete the data of supplementary materials Figure S2 in the first version. This previous Figure S2 has been reordered as new Figure S5. It was in the last separate page of the supplementary materials. I guess this reviewer may miss it. For the requested data of how the silencing of HDAC5 expression affects MEF2 and HDAC9 expression, with and without Ang-II stimulation, we have already shown these data in the new Figure S2 (not original Figure S2, which has been re-ordered as Figure S5).
